# Enzyme inclusion or fermentation of canola-based diets generate different responses in growth indicators, carcass quality, nutrient digestibility, bone strength, and blood biochemical parameters in broiler chickens

**DOI:** 10.5194/aab-68-485-2025

**Published:** 2025-07-25

**Authors:** Abdul Hafeez, Waseem Akram, Hanan Al-Khalaifah, Shabana Naz, Rifat Ullah Khan, Vincenzo Tufarelli, Ibrahim A. Alhidary

**Affiliations:** 1 Department of Poultry Science, Faculty of Animal Husbandry and Veterinary Sciences, The University of Agriculture, Peshawar, Peshawar, Pakistan; 2 Environment and Life Sciences Research Center, Kuwait Institute for Scientific Research, Kuwait City, Kuwait; 3 Department of Zoology, Government College University Faisalabad, Faisalabad, Pakistan; 4 Department of Animal Production, College of Agriculture and Food Sciences, King Saud University, Riyadh, Saudi Arabia; 5 Physiology Lab, College of Veterinary Sciences, Faculty of Animal Husbandry and Veterinary Sciences, The University of Agriculture, Peshawar, Peshawar, Pakistan; 6 Department of Precision and Regenerative Medicine and Jonian Area (DiMePRe-J), Section of Veterinary Science and Animal Production, University of Bari “Aldo Moro”, s.p. Casamassima km 3, 70010 Valenzano, Italy

## Abstract

This study aimed to evaluate the effects of dietary enzyme and fermentation supplementation on the growth performance, carcass traits, nutrient digestibility, and bone and blood characteristics of broiler chickens. A total of 900 male Hubbard broilers were assigned to three treatment groups: a control diet, fermented canola meal (6 %, 12 %, and 18 %), and enzyme-treated canola meal (6 %, 12 %, and 18 %). The results showed that both enzyme treatments resulted in superior weight gain, while 18 % fermentation supplementation had negative effects on weight gain and feed conversion ratio (FCR). Carcass traits, including dressing percentage and eviscerated weight, were significantly higher in enzyme-treated and fermented groups. Nutrient digestibility, particularly of crude protein and crude fiber, was improved with fermentation supplementation, with the best results being observed at 6 % and 12 % levels. Bone characteristics such as bone weight enhancement, robusticity index, and tibio-tarsal index were decreased ( P<0.01 ) in fermented-diet-fed birds. Blood biochemical analysis revealed reduced triglyceride levels in broilers fed with a fermented diet, while other parameters, including cholesterol and glucose, remained unaffected. These findings suggest that optimal levels of fermentation and enzyme supplementation can enhance broiler productivity and health.

## 1 Introduction

The escalating costs of conventional protein sources like soybean meal (SBM) in poultry diets have spurred interest in alternative protein ingredients (Ajmal et al., 2023; Abudabos et al., 2017; Hafeez et al., 2025), including canola meal (CM). Canola meal offers a high crude protein content (35 %–40 %) and a significant amount of sulfur-containing amino acids, making it a promising substitute for SBM (Velayudhan et al., 2018; Boroojeni et al., 2022). However, its use is constrained by the presence of anti-nutritional factors such as glucosinolates, tannins, phytates, and crude fiber, which impair nutrient digestibility and overall feed efficiency in poultry (Kocher et al., 2003; Newkirk, 2009).

Fermentation and enzyme supplementation have emerged as effective strategies to mitigate the effects of these anti-nutritional factors. Fermentation with probiotic bacteria such as *Lactobacillus fermentum* and *Bacillus subtilis* enhances the nutritional profile of CM by reducing glucosinolate content, increasing digestibility, and improving the bioavailability of essential amino acids and peptides (Feng et al., 2007; Jakobsen et al., 2015; Elbaz et al., 2023; Sultan et al., 2024a, b; Yu et al., 2024). Furthermore, fermentation enhances gut health by promoting beneficial microbiota and reducing harmful pathogens like *Escherichia coli* (Elbaz et al., 2023; Wang et al., 2024). Solid-state fermentation (SSF) using microorganisms reduces anti-nutrients (Omar et al., 2021), enhances digestibility, and enriches nutrients like peptides and vitamins, though their efficacy in broiler diets needs further study (Drazbo et al., 2019; Guo et al., 2020; Zaworska-Zakrzewska et al., 2023; Wardah et al., 2023).

Enzyme supplementation complements fermentation by degrading non-starch polysaccharides (NSPs), thereby improving nutrient absorption and reducing gastrointestinal viscosity (Cowieson, 2010; Hafeez et al., 2021; Ahsan et al., 2024a, b; Khan et al., 2024). Exogenous enzymes such as proteases, amylases, and phytases enhance the bioavailability of nutrients and minimize nutrient excretion, reducing environmental pollution (Jabbar et al., 2021a, b; Alqahtani et al., 2024; Sultan et al., 2024a, b). Studies demonstrate that the combination of fermentation and enzyme supplementation in broiler diets optimizes growth performance, improves feed conversion ratios (FCRs), enhances immune responses and antioxidant capacity, and improves gut morphology (Elbaz et al., 2023).

In light of these findings, the current research work evaluates the synergistic effects of dietary enzymes and fermentation supplementation on the growth indicators, carcass quality, nutrient digestibility, bone strength, and blood biochemical parameters of broiler chickens consuming canola-based meal. This approach underscores the potential of fermented and enzyme-supplemented CM as a sustainable, cost-effective alternative to SBM, contributing to more efficient and environmentally friendly poultry production systems.

## 2 Materials and methods

### 2.1 Experimental design, birds, and diets

A total of 900 1 d old male Hubbard broiler chicks, obtained from a local hatchery, were used in this study. The chicks, with an initial average body weight of 44.53 ± 0.05 g, were randomly allocated to three dietary treatment groups, each consisting of five replicates. The dietary treatments included the following:

1. *Control group.* Birds were fed a standard canola basal diet without any supplementation of enzymes or fermented ingredients.
2. *Fermented canola meal group.* Birds received diets containing fermented canola meal at graded levels of 6 %, 12 %, and 18 %.
3. *Enzyme-treated canola meal group.* Birds were provided diets incorporating enzyme-treated canola meal at the same inclusion rates of 6 %, 12 %, and 18 %.

The experiment followed a 3×3 factorial design, incorporating three levels of enzyme treatment and three levels of fermentation supplementation. All experimental diets were formulated to fulfill the nutritional requirements of broiler chickens throughout the trial period (refer to Tables 1 and 2). Birds had unrestricted access to feed and clean drinking water for the entire 35 d duration of the study. Standardized broiler husbandry protocols were adhered to, encompassing optimal lighting, temperature regulation, and strict biosecurity to reduce environmental variation and to support uniform growth. The chicks were housed in a well-insulated, hygienic facility with sufficient ventilation to ensure thermal comfort. Brooding temperatures were maintained between 32 and 34 °C during the initial week and then were decreased incrementally by 2–3 °C per week, stabilizing at 24–26 °C by the conclusion of the trial. A continuous 24 h light schedule was implemented for the first 3 d post-hatch to facilitate chick adaptation and to encourage feeding and drinking behavior. Thereafter, a photoperiod of 20 h of light and 4 h of darkness per day was applied. Bedding composed of materials such as wood shavings or rice husks was maintained at a thickness of 5–7 cm to promote insulation and moisture absorption. The litter condition was routinely monitored and managed by stirring or replacing it to maintain dryness and to suppress microbial proliferation.

**Table 1:** Feed composition of basal diet for starter phase (1–21 d) and finisher phase (22–35 d).

Ingredients^1^	Starter diet (% canola meal)			Finisher diet (% canola meal)		
	6 %	12 %	18 %	6 %	12 %	18 %
Corn	61.27	64.16	64.05	71.85	74.58	70.10
Soybean meal	15.14	11.35	8.11	7.98	4.31	1.73
Rice polishing	8.05	3.18	0.00	5.28	0.00	0.00
Canola meal	6.00	12.0	18.0	6.0	12.0	18.0
PBM / APC high fat	4.00	4.0	4.0	5.00	5.0	5.0
Limestone / chips	0.98	0.91	0.84	0.74	0.65	0.56
Lysine sulfate	0.83	0.87	0.88	0.86	0.90	0.87
MCP	0.82	0.80	0.77	0.00	0.00	0.00
L-valine	0.60	0.60	0.59	0.19	0.19	0.17
L-methionine	0.38	0.35	0.32	0.34	0.32	0.29
Salt	0.31	0.31	0.30	0.17	0.17	0.16
L-arginine	0.31	0.34	0.35	0.36	0.39	0.38
Potassium carbon	0.30	0.10	0.18	0.25	0.37	0.36
L-threonine	0.27	0.27	0.26	0.24	0.24	0.22
L-isoleucine	0.17	0.18	0.18	0.19	0.20	0.19
Sodium bicarbonate	0.15	0.15	0.15	0.15	0.15	0.15
Toxin binder	0.08	0.08	0.08	0.05	0.05	0.05
Vitamin premix	0.05	0.08	0.05	0.05	0.05	0.05
Choline chloride	0.05	0.05	0.05	0.05	0.05	0.05
Mineral premix	0.05	0.05	0.05	0.05	0.05	0.05
Betaine	0.05	0.05	0.05	0.05	0.05	0.05
L-tryptophan	0.02	0.02	0.02	0.06	0.06	0.05
Antioxidant	0.02	0.02	0.02	0.01	0.01	0.01
Enramycin 4 %	0.0100	0.01	0.01	0.01	0.01	0.01
Diclazuril 1 %	0.0100	0.01	0.01	0.00	0.00	0
Axtra PHY GOLD 1	0.0100	0.0100	0.01	0.01	0.0100	0.01
Total	100	100	100	100	100	100
Chemical analysis	Starter phase			Finisher phase		
	6 %	12 %	18 %	6 %	12 %	18 %
Dry matter	88.70	88.67	88.74	88.555	88.578	88.702
Moisture	11.29	11.32	11.26	11.445	11.422	11.298
ME ( $\mathrm{kcal}\;{\mathrm{kg}}^{-1}$ )	2900.00	2900.00	2900.0	3050.000	3050.000	3050.000
CP	19.23	19.38	19.56	16.868	16.954	17.420
Ash	5.67	5.25	5.17	4.156	3.963	4.085
Fat	4.73	4.26	4.52	4.791	4.401	5.651
Fiber	3.29	3.49	3.78	3.009	3.183	3.635

^1^ Previously undefined abbreviations used in the table are as follows: PBM – poultry byproduct meal; APC – animal protein concentrate; MCP – monocalcium phosphate; CP – crude protein. Provided per kilogram of diet: vitamin A, 10 000 IU; vitamin E, 30 IU; vitamin D3,0000 IU; thiamine, 2.5 mg; vitamin K3, 2.5 mg; pyridoxine, 5 mg; riboflavin, 5.5 mg; folic acid, 0.7 mg; vitamin B12, 0.5 mg; pantothenic acid, 15 mg; nicotinic acid, 50 mg; biotin, 0.2 mg; copper, 12 mg; manganese, 100 mg; iron, 95 mg; selenium, 0.5 mg; iodine, 0.5 mg; zinc, 100 mg.

**Table 2:** Main treatment effect of dietary enzyme treatment and fermentation on feed intake (g), weight gain (g), and FCR in broilers.

	Treatments			Levels			SEM	P values		
	Untreated	Enzyme treated	Fermented	6 %	12 %	18 %		Treatment	Level	T×L
Feed intake	3456	3454	3454	3550^a^	3420^b^	3394^b^	18.59	1.00	<0.01	0.23
Weight gain	1749^b^	1793^a^	1802^a^	1836^a^	1832^a^	1676^b^	18.3	0.01	<0.01	<0.01
FCR	1.98	1.93	1.92	1.93^b^	1.87^b^	2.03^a^	0.02	0.072	<0.01	<0.01

Means in the same row with different superscripts are significantly different ( P<0.05 ).

### 2.2 Enzyme treatment

Canola meal was ground and mixed with water at a 1:1 ratio at ambient temperature, followed by the addition of three specific enzyme formulations. These included Axtra PHY (200 $g\;t^{-1}$ ; DuPont, Wilmington, DE, USA), a phytase-based product; Axtra PRO (50 $g\;t^{-1}$ ; DuPont, Wilmington, DE, USA), which provides protease activity; and Hemicell (300 $g\;t^{-1}$ ; Elanco, Indiana, USA), containing β -mannanase. The water used for soaking was acidified with an organic acid blend of Silo Health (1 $\mathrm{kg}\;t^{-1}$ ; Silo, China). The enzyme incubation process was conducted at 30 °C for 24 h to ensure adequate enzymatic action. After incubation, the treated material was subjected to rapid drying – completed in under 3 s – to preserve nutrient integrity. The resulting pre-treated canola meal was incorporated into experimental diets at inclusion levels of 6 %, 12 %, and 18 % by replacing portions of a standard basal diet.

### 2.3 Solid-state fermentation of canola meal

Canola meal was ground and combined with water at a 1:1 ratio, followed by inoculation with CLOSTAT (Kemin, USA), a commercial probiotic product containing the registered Bacillus subtilis strain PB6. The mixture was fermented under controlled conditions for 48 h at a temperature range of 25 to 37 °C to facilitate effective microbial fermentation. After the fermentation period, the material was dried until the moisture content was reduced to 10 %–12 %, ensuring product stability during storage. The resulting fermented canola meal was then added to experimental diets at inclusion levels of 6 %, 12 %, and 18 % to assess its nutritional improvements.

### 2.4 Evaluation of growth performance

The performance metrics assessed in this experiment included total feed intake, body weight gain, and feed conversion ratio (FCR). Feed intake was recorded as the cumulative amount of feed consumed throughout the study duration. Body weight gain was calculated by subtracting the initial weight from the final weight of the birds. The feed conversion ratio was derived by dividing the total feed intake by the corresponding weight gain, serving as a measure of how efficiently the feed was utilized for growth.

### 2.5 Carcass traits

For carcass quality traits, two birds were randomly selected from each replicate on day 35 of the study. The dressed carcass weight (excluding feathers, blood, and viscera) was recorded and expressed as a percentage of live body weight. The eviscerated carcass weight (excluding the gastrointestinal tract, liver, and other internal organs) was determined and expressed as a percentage of live body weight. The combined weight of the liver, heart, and gizzard was recorded and expressed in grams. The abdominal fat, including fat deposits around the gizzard and cloacal region, was carefully dissected and weighed. Meat pH was measured with the help of a digital pH meter (Hanna Instruments, USA).

### 2.6 Determination of apparent total nutrient digestibility

On day 31 of the trial, five birds per replicate were randomly selected and transferred to metabolic cages for a 4 d fecal collection phase. Feed intake and fecal output were measured daily to determine apparent total nutrient digestibility. Post-euthanasia, the ileum was excised, immediately chilled, and prepared for subsequent chemical analysis. The ileal contents were freeze-dried and subjected to laboratory analysis for dry matter, crude protein, ether extract, crude fiber, and apparent metabolizable energy following the procedures described by Ramaiyulis et al. (2023).

$$\begin{array}{rl} \text{Apparent } & \text{total digestibility (\%)}=\\ & \frac{\text{Conc. of marker in diet}}{\text{Conc. of marker in digesta}}\times \\ & \frac{\text{Conc. of nutrient in digesta}}{\text{Conc. of nutrient in diet}}\times 100 \end{array}$$

### 2.7 Bone quality

On day 35, two birds per replicate were selected. The birds were slaughtered with a knife, and the left legs were removed from each bird. After removing the patella and meat from the legs, the left tibia bone was collected, thoroughly washed, and weighed. The bone length was measured, and the lateral and medial wall thicknesses were determined at the midpoint. The ratio of the body weight to the bone weight was calculated by dividing the body weight by the bone weight. The following indices were determined: 

Robusticity index = bone length / cube root of bone weight (Yu et al., 2024). 

Tibio-tarsal index = (diaphysis diameter - medullary canal diameter) / diaphysis diameter × 100 (Barnett and Nordin, 1990). 

Subsequently, the bone was dried in an oven at 100 °C for 24 h to determine dry weight, while ash content was measured at 550 °C using a muffle furnace.

### 2.8 Blood biochemistry

On day 35 of the experiment, two birds per replicate were selected and slaughtered with a sharp knife, and their serum was analyzed using a biochemistry analyzer (ChemWell^®^ 2910, Awareness Technology Inc., USA) to measure total protein, triglycerides, high-density lipoprotein (HDL), low-density lipoprotein (LDL), total cholesterol, and glucose (Asghar et al., 2024; Kalsoom et al., 2024).

### 2.9 Statistical analysis

The study followed a 3×3 factorial arrangement within a completely randomized design (CRD). Statistical evaluations were conducted to determine the individual and interactive effects of canola meal processing methods (untreated, enzyme treated, and fermented) and inclusion levels (3 %, 6 %, and 9 %). Data were subjected to an analysis of variance (ANOVA) using SPSS software (version 21.0). The pen served as the experimental unit for all analyses. Treatment means were compared using Tukey's post hoc test when significant differences were detected at P<0.05 .

## 3 Results

Table 2 presents the effects of dietary supplementation of enzyme and fermentation levels (6 %, 12 %, and 18 %) on feed intake, weight gain, and feed conversion ratio (FCR) in broilers chickens. The analysis shows no significant differences in terms of feed intake across treatments (untreated, enzyme treated, and fermented), with values being consistently around 3454–3456 g. However, feed intake varied significantly with fermentation supplementation levels, with the highest intake being recorded at 6 % (3550 g) compared to lower intakes at 12 % (3420 g) and 18 % (3394 g) ( P<0.01 ).

Weight gain was significantly influenced by both treatments and fermentation levels. Enzyme-treated and fermented groups outperformed the untreated group, with gains ranging from 1793 to 1802 g, while the untreated group recorded 1749 g. Lower levels of fermentation supplementation (6 % and 12 %) produced better weight gains (1836 and 1832 g) compared to the 18 % level (1676 g) ( P<0.01 ).

Although no significant differences in terms of FCR were observed across treatments, fermentation supplementation at 18 % resulted in a poorer FCR (2.03) compared to 6 % (1.93) and 12 % (1.87) ( P<0.01 ). Statistical analysis indicates significant treatment and level effects, with interactions being observed for weight gain and FCR.

Table 3 shows the effects of dietary enzyme treatment and fermentation level supplementation (6 %, 12 %, and 18 %) on carcass characteristics in broilers. The dressing percentage was significantly higher for enzyme-treated (66.8 %) and fermented (67.1 %) broilers compared to the untreated group (64.4 %) ( P<0.01 ). Fermentation level supplementation also influenced dressing percentage, with 6 % and 12 % achieving higher values (67.2 % and 66.7 %, respectively) compared to 18 % (64.4 %).

**Table 3:** Main treatment effect of dietary enzyme treatment and fermentation on carcass characteristics in broilers.

Treatment	Treatment			Level			SEM	P values		
Level	Untreated	Enzyme	Fermented	6 %	12 %	18 %		Treatment	Level	T×L
		treated								
Dressing %	64.4^b^	66.8^a^	67.1^a^	67.2^a^	66.7^a^	64.4^b^	0.52	<0.01	<0.01	<0.01
Eviscerated weight (%)	73.5^b^	76.9^a^	80.2^a^	79.1^a^	77.6^a^	74.0^b^	0.85	<0.01	<0.01	0.29
Giblet weight (g)	82.7	78.0	79.00	76.4^b^	86.2^a^	77.0^ab^	1.82	0.44	0.03	0.12
Abdominal fat weight (g)	1.75	1.69	1.63	1.58^b^	1.66^ab^	1.83^a^	0.04	0.42	0.04	0.92
Meat pH	5.97	5.98	5.97	5.98	5.97	5.97	0.01	0.88	0.88	0.97

Means in the same row with different superscripts are significantly different ( P<0.05 ).

Eviscerated weight followed a similar pattern, with higher percentages in enzyme-treated (76.9 %) and fermented (80.2 %) groups compared to in untreated broilers (73.5 %) ( P<0.01 ). Fermentation levels of 6 % and 12 % supplementation yielded better eviscerated weights (79.1 % and 77.6 %) compared to 18 % (74.0 %).

Giblet weight was not significantly affected by treatments, although fermentation supplementation at 12 % resulted in the highest weight (86.2 g) compared to 18 % (77.0 g) and 6 % (76.4 g) ( P=0.03 ). Abdominal fat weight was not significantly affected by treatments, though the highest fat weight (1.83 g) was observed at the 18 % fermentation level supplementation, contrasting with lower values at 6 % (1.58 g) ( P=0.04 ). Meat pH remained stable across treatments and fermentation level supplementations, with no significant differences ( P>0.05 ). Different superscripts indicate significant differences at P<0.05 .

Table 4 summarizes the effects of dietary enzyme treatment and fermentation level supplementation (6 %, 12 %, and 18 %) on the apparent total digestibility (ATD) of nutrients during the finisher phase in broilers.

**Table 4:** Main treatment effect of dietary enzyme treatment and fermentation on percentage apparent total digestibility (ATD) of nutrients at finisher phase in broilers.

	Treatment			Level			SEM	P values		
	Untreated	Enzyme	Fermented	6 %	12 %	18 %		Treatment	Level	T×L
		treated								
DM	72.9	72.9	73.9	72.0	74.0	73.6	0.43	0.55	0.16	0.31
ASH	46.6	47.9	47.8	47.6	48.3	46.5	0.41	0.36	0.23	0.98
CP	63.7^b^	65.1^b^	69.5^a^	66.9^a^	69.2^a^	62.1^b^	0.99	<0.01	<0.01	<0.01
CF	72.6^b^	72.6^b^	77.4^a^	76.0^a^	76.5^a^	70.1^b^	0.90	<0.01	<0.01	<0.01
NFE	80.8^b^	81.3^b^	83.8^a^	82.3^ab^	83.6^a^	80.1^b^	0.57	0.01	<0.01	<0.01
Ca	25.8^b^	27.7^a^	26.6^ab^	26.4^b^	28.2^a^	25.5^b^	0.43	0.04	<0.01	<0.01
P	25.8	26.1	26.0	26.3^ab^	28.0^a^	23.7^b^	0.58	0.97	<0.01	0.01

Means in the same row with different superscripts are significantly different at α=0.05 .

Dry matter (DM) and ash digestibility were not significantly influenced by treatments or fermentation level supplementation, with values ranging from 72.0 % to 74.0 % for DM and 46.5 % to 48.3 % for ash ( P>0.05 ).

Crude protein (CP) digestibility was significantly higher in the fermented-diet-fed broilers (69.5 %) compared to in the untreated (63.7 %) and enzyme-treated (65.1 %) groups ( P<0.01 ). Fermentation levels at 6 % and 12 % supplementation produced superior CP digestibility (66.9 % and 69.2 %) compared to 18 % (62.1 %) ( P<0.01 ).

Crude fiber (CF) and nitrogen-free extract (NFE) digestibility followed similar trends, with fermentation level supplementation significantly improving digestibility (77.4 % for CF and 83.8 % for NFE) compared to in untreated and enzyme-treated broilers ( P<0.01 ). Calcium (Ca) and phosphorus (P) digestibility showed moderate improvements, with the highest Ca digestibility at 12 % (28.2 %) and the highest P digestibility at 12 % (28.0 %).

Table 5 highlights the effects of dietary enzyme treatment and fermentation level supplementation (6 %, 12 %, and 18 %) on bone characteristics in broilers during the finisher phase.

**Table 5:** Main treatment effect of dietary enzyme treatment and fermentation on bone finisher canola treatment in broilers.

Treatment	Treatment			Level			SEM	P values		
Level	Untreated	Enzyme	Fermented	6 %	12 %	18 %		Treatment	Level	T×L
		treated								
Bone weight (g)	6.41^b^	6.65^b^	8.13^a^	7.74^a^	7.60^a^	5.84^b^	0.31	<0.01	<0.01	0.04
Bone length (cm)	91.0	90.2	86.7	89.8	88.3	89.8	0.76	0.06	0.64	0.55
BW : bone wt	250	252	252	251	252	250	1.54	0.86	0.89	0.34
Robusticity index	4.93^a^	4.84^a^	4.36^b^	4.57^b^	4.54^b^	5.03^a^	0.08	<0.01	<0.01	0.37
Tibio-tarsal index	39.9^a^	36.7^ab^	31.4^b^	38.0	34.9	35.2	1.26	0.02	0.49	0.59

Means in the same row with different superscripts are significantly different ( P<0.05 ).

Bone weight was significantly higher in fermented-diet-fed broilers (8.13 g) compared to in untreated (6.41 g) and enzyme-treated (6.65 g) groups ( P<0.01 ). Fermentation levels of 6 % and 12 % supplementation yielded higher bone weights (7.74 and 7.60 g, respectively) compared to 18 % (5.84 g) ( P<0.01 ).

Bone length showed no significant treatment or level effects, with values ranging from 86.7 to 91.0 cm ( P>0.05 ). The ratio of bone weight to body weight (BW : bone wt) remained consistent across treatments and levels.

The robusticity index was significantly lower in fermented-diet-fed broilers (4.36) compared to in enzyme-treated and untreated groups (4.93 and 4.84, respectively) ( P<0.01 ). The tibio-tarsal index followed a similar trend, with fermented-diet-fed broilers showing lower values (31.4) compared to untreated broilers (39.9) ( P<0.05 ). Different superscripts indicate significant differences at P<0.05 .

Table 6 summarizes the effects of dietary enzyme treatment and fermentation level supplementation (6 %, 12 %, and 18 %) on blood biochemical parameters in broilers during the finisher phase.

**Table 6:** Main treatment effect of dietary enzyme treatment and fermentation on blood biochemical parameters at finisher phase in broilers.

	Treatment			Level			SEM	P values		
	Untreated	Enzyme	Fermented	6 %	12 %	18 %		Treatment	Level	T×L
		treated								
Triglycerides ( $\mathrm{mg}\;{\mathrm{dL}}^{-1}$ )	41.5^a^	40.2^ab^	38.2^b^	40.4	39.9	39.7	0.45	0.01	0.75	0.31
LDL ( $\mathrm{mg}\;{\mathrm{dL}}^{-1}$ )	37.9	39.1	39.9	39.9	39.3	37.8	0.72	0.55	0.47	0.32
HDL ( $\mathrm{mg}\;{\mathrm{dL}}^{-1}$ )	56.3	56.9	58.2	57.4	56.4	57.6	0.71	0.50	0.69	0.04
Total cholesterol ( $\mathrm{mg}\;{\mathrm{dL}}^{-1}$ )	103	104	106	105	104	103	0.91	0.43	0.64	0.70
Blood glucose ( $\mathrm{mg}\;{\mathrm{dL}}^{-1}$ )	111.7	111.7	111.8	111.5	111.9	111.8	0.93	0.94	0.57	0.66
Total protein ( $g\;{\mathrm{dL}}^{-1}$ )	4.26	4.19	4.33	4.28	4.32	4.18	0.12	0.91	0.91	0.64

Means in the same row with different superscripts are significantly different ( P<0.05 ).

Triglyceride levels were significantly lower in fermented-diet-fed broilers (38.2 $\mathrm{mg}\;{\mathrm{dL}}^{-1}$ ) compared to in the untreated group (41.5 $\mathrm{mg}\;{\mathrm{dL}}^{-1}$ ) ( P=0.01 ). However, fermentation level supplementation did not produce significant changes ( P>0.05 ). LDL levels showed no significant differences among treatments or levels, remaining within a narrow range of 37.8 to 39.9 $\mathrm{mg}\;{\mathrm{dL}}^{-1}$ ( P>0.05 ).

HDL levels exhibited no significant difference across treatments or level effects, although fermented-diet-fed broilers showed a slight increase (58.2 $\mathrm{mg}\;{\mathrm{dL}}^{-1}$ ). Total cholesterol, blood glucose, and total protein levels remained consistent across all treatments and fermentation level supplementations, with no statistically significant variations ( P>0.05 ).

These results indicate limited effects of dietary enzyme treatment and fermentation level supplementation on most blood biochemical parameters, with the notable exception of triglyceride reduction in fermented-diet-fed broilers.

## 4 Discussion

The study highlights the significant impact of dietary enzyme treatment and fermentation supplementation on broiler growth performance. The findings align with previous research indicating that enzyme supplementation and fermentation enhance nutrient utilization and improve feed conversion ratios (FCRs) in poultry (Xu et al., 2012; Aini et al., 2023; Mohamed et al., 2023). The observed higher weight gains in groups receiving enzyme-treated and fermented canola meal groups support the findings of Elbaz et al. (2021) and Saeed et al. (2025), who reported improved feed efficiency and body weight when broilers were fed a fermented canola meal diet.

The highest feed intake at the 6 % fermentation level supplementation in this study may be attributed to better palatability and enhanced nutrient availability, consistently with Xu et al. (2012), who found that fermentation improves nutrient digestibility and reduces anti-nutritional factors. Conversely, the lower feed intake at the 18 % level may indicate palatability issues or potential adverse effects of high fermentation concentrations, as previously noted in studies on excessive fermentation levels. The findings from Toghyani et al. (2017) also suggest that canola meal diets with higher fiber and sulfur content could negatively influence feed intake and body weight gain due to increased gizzard fill and reduced palatability.

Although FCR values showed no significant variation across treatments, the poorer FCR observed at the 18 % fermentation level supplementation suggests an inefficiency in nutrient utilization, potentially due to imbalanced dietary components or reduced feed digestibility (Xu et al., 2012; Elbaz et al., 2021). Similar trends were observed by Toghyani et al. (2017), where high levels of canola meal reduced nutrient digestibility, increased digesta viscosity, and negatively impacted protein and energy digestibility. The reduced metabolizable energy (ME) and net energy (NE) values observed in high-fiber diets emphasize the importance of optimizing fermentation level supplementation to maximize energy utilization without impairing growth performance (Susalam et al., 2024).

Enzyme supplementation plays a crucial role in counteracting the negative effects of high-fiber diets (Anwar et al., 2023a, b). Toghyani et al. (2017) demonstrated that carbohydrase supplementation significantly improved NE by reducing heat production and increasing retained energy, aligning with the present findings on non-significantly enhanced efficiency in enzyme-treated groups. Protease supplementation also improved protein digestibility, supporting earlier studies showing that exogenous proteases enhance amino acid availability by breaking down encapsulated protein structures (Cowieson et al., 2010). Overall, the results emphasize the need for tailored feeding strategies to harness the benefits of enzyme treatment and fermentation supplementation in broiler diets while avoiding excessive concentrations. Optimizing fermentation and enzyme supplementation is essential to maximize nutrient utilization and growth performance in broilers fed alternative protein sources such as canola meal.

The results indicate that dietary enzyme treatment and fermentation supplementation significantly influenced carcass characteristics in broilers, aligning with previous findings on the benefits of enzyme supplementation and microbial fermentation in poultry nutrition (Toghyani et al., 2017; Elbaz et al., 2021).

The significantly higher dressing percentage in enzyme-treated (66.8 %) and fermented (67.1 %) broilers compared to in untreated birds (64.4 %) suggests improved nutrient utilization and growth efficiency. Toghyani et al. (2017) demonstrated that enzyme supplementation enhances digestibility and energy retention, leading to better carcass yields. The positive effect of fermentation supplementation, particularly at 6 % and 12 %, may be attributed to improved feed digestibility and reduced anti-nutritional factors, as previously reported by Xu et al. (2012). The lower dressing percentage at untreated and 18 % fermentation (64.4 %) may indicate diminishing returns due to excessive fermentation, potentially reducing nutrient availability or causing palatability issues (Disetlhe et al., 2019).

Eviscerated weight followed a similar trend, with enzyme treatment (76.9 %) and fermentation supplementation (80.2 %) significantly improving yields. This aligns with findings from high-canola-meal diets where enzyme supplementation improved nutrient absorption and energy efficiency, contributing to better carcass quality (Toghyani et al., 2017). The superior eviscerated weight at 6 % and 12 % fermentation level supplementation (79.1 % and 77.6 %) supports the hypothesis that moderate fermentation level supplementation (6 % and 12 %) enhances digestion, while 18 % fermentation supplementation (74.0 %) may negatively impact feed efficiency, as observed in previous studies on excessive fermentation (Elbaz et al., 2021).

Giblet weight was not significantly affected by enzyme supplementation, but fermentation at 12 % supplementation resulted in the highest giblet weight (86.2 g), suggesting optimal organ development at this level. Similar trends have been reported in studies where fermentation improved gut health and organ function (Cowieson et al., 2010). The lower giblet weight at 18 % fermentation supplementation (77.0 g) may reflect a negative impact of excessive fermentation on organ growth, possibly linked to increased metabolic demands for detoxification of fermentation byproducts (Disetlhe et al., 2019).

Abdominal fat weight remained unaffected by treatments, though the highest fat accumulation was observed at 18 % fermentation supplementation (1.83 g). This finding aligns with previous research indicating that excessive fermentation can alter lipid metabolism, potentially leading to increased fat deposition (Xu et al., 2012). The lower fat weights at 6 % and 12 % fermentation supplementation (1.58 g) suggest better energy partitioning for muscle growth rather than fat storage, consistently with improved FCRs observed under optimal fermentation levels (Toghyani et al., 2017).

Meat pH stability across treatments indicates that neither enzyme supplementation nor fermentation significantly influenced post-mortem muscle acidification, which is crucial for meat quality and shelf life. This stability suggests that dietary modifications did not induce stress-related metabolic shifts that could impact meat pH, aligning with findings in enzyme-treated and fermented diets in broilers (Elbaz et al., 2021).

Overall, these results emphasize the importance of optimizing fermentation levels and enzyme supplementation to enhance carcass traits. Moderate fermentation (6 %–12 %) and enzyme inclusion effectively improved dressing percentage and eviscerated weight, whereas excessive fermentation supplementation (18 %) showed diminishing benefits and potential drawbacks in nutrient utilization and fat deposition.

The lack of significant differences in terms of DM and ash digestibility across treatments, with values ranging from 72.0 % to 74.0 % for DM and 46.5 % to 48.3 % for ash, suggests that these components are less responsive to enzyme supplementation or fermentation processes. This aligns with previous research indicating that certain nutrients may not exhibit enhanced digestibility despite dietary modifications (Bach Knudsen et al., 1997). The significant improvement in CP digestibility in fermented-diet-fed broilers compared to in untreated (63.7 %) and enzyme-treated (65.1 %) groups underscores the efficacy of fermentation supplementation in enhancing protein utilization. Fermentation processes degrade anti-nutritional factors and increase amino acid availability, thereby improving protein digestibility. Notably, fermentation supplementation at 6 % and 12 % yielded superior CP digestibility (66.9 % and 69.2 %, respectively) compared to at 18 % (62.1 %), indicating that moderate fermentation levels are optimal, while higher levels may not confer additional benefits.

The observed enhancements in CF and NFE digestibility in fermented groups (77.4 % for CF and 83.8 % for NFE) align with findings that fermentation can break down complex carbohydrates and fibers, facilitating better nutrient absorption. This improvement is consistent with studies demonstrating that fermentation reduces fiber complexity, thereby enhancing digestibility (Akram et al., 2023).

The moderate improvements in Ca and P digestibility, with the highest values being observed at 12 % fermentation supplementation (28.2 % for Ca and 28.0 % for P), suggest that fermentation at this level may enhance mineral availability. This is possibly due to the degradation of phytates during fermentation, which increases mineral bioavailability. However, the lack of significant effects at other fermentation levels indicates that the relationship between fermentation and mineral digestibility is complex and may require further investigation (Hu et al., 2016).

The significantly higher bone weight in fermented-diet-fed broilers (8.13 g) compared to in untreated (6.41 g) and enzyme-treated (6.65 g) groups suggests that fermentation supplementation may enhance mineral retention and bone accretion. This trend is further reinforced by the highest bone weights being observed at 6 % and 12 % fermentation level supplementation (7.74 and 7.60 g, respectively) compared to at 18 % (5.84 g), indicating that excessive fermentation may not sustain bone mineralization. The mechanism behind these improvements could be linked to the enhanced phosphorus (P) and calcium (Ca) digestibility observed in fermented diets (Hafeez et al., 2025), essential for bone development. Additionally, microbial fermentation can degrade phytic acid, increasing mineral bioavailability (Ali et al., 2023), potentially supporting greater bone mass.

Bone length remained unaffected across treatments, with values ranging from 86.7 to 91.0 cm, suggesting that dietary modifications influence bone density and composition rather than longitudinal growth. The stable BW : bone wt ratio further supports the notion that fermentation enhances bone mineralization without altering proportional skeletal development.

The significantly lower robusticity index in fermented-diet-fed broilers (4.36) compared to in enzyme-treated (4.93) and untreated groups (4.84) suggests a shift in bone architecture favoring increased mineral density over structural elongation. Similarly, the lower tibio-tarsal index in fermented-diet-fed broilers (31.4) compared to in untreated broilers (39.9) ( P<0.05 ) indicates a potential reduction in bone porosity or an increase in compact bone formation, which may be beneficial for skeletal integrity. While no prior studies have specifically examined these indices in response to dietary fermentation, it is well-established that improved mineral utilization enhances bone strength and structural properties (Hu et al., 2016).

In the current study, the significantly lower triglyceride levels in fermented-diet-fed broilers (38.2 $\mathrm{mg}\;{\mathrm{dL}}^{-1}$ ) compared to in untreated birds (41.5 $\mathrm{mg}\;{\mathrm{dL}}^{-1}$ ) suggest that fermentation supplementation may enhance lipid metabolism. This aligns with findings from Elbaz et al. (2021), who reported reduced cholesterol and triglycerides in broilers fed fermented canola meal, likely due to improved lipid digestion and utilization. Fermentation has been shown to increase beneficial gut microflora, such as *Lactobacillus* spp., which can influence lipid metabolism by modulating bile acid metabolism and reducing lipid absorption (Elbaz et al., 2021).

The absence of significant effects on LDL, HDL, and total cholesterol is consistent with previous reports indicating that, while fermentation can modulate triglyceride levels, its effects on overall cholesterol metabolism remain inconsistent (Hu et al., 2016). Similarly, Elbaz et al. (2021) observed a non-significant increase in HDL levels in broilers fed fermented diets, which aligns with the slight but non-significant increase in HDL (58.2 $\mathrm{mg}\;{\mathrm{dL}}^{-1}$ ) observed in the present study.

The stable blood glucose and total protein levels across treatments suggest that fermentation and enzyme supplementation did not substantially alter energy metabolism or protein utilization. This finding is consistent with previous research indicating that, while fermentation improves nutrient digestibility, its effects on systemic blood metabolites may be minimal (Hu et al., 2016).

## 5 Conclusion

Dietary enzyme supplementation and fermentation supplementation at 6 % and 12 % improved growth performance, carcass traits, nutrient digestibility, and bone characteristics in broilers, while excessive fermentation supplementation (18 %) negatively affected these parameters. Fermentation supplementation also reduced triglyceride levels but had minimal impact on other blood biochemical parameters, highlighting the need for optimal fermentation levels to maximize broiler productivity. In practice, incorporating enzyme-treated or fermented canola meal at 6 %–12 % is recommended to enhance broiler performance, while higher levels should be avoided due to negative effects. Future research should explore the mechanisms behind the reduced efficacy at higher inclusion rates and assess the long-term impacts on health and meat quality.

## Supplement

10.5194/aab-68-485-2025-supplement The supplement related to this article is available online at https://doi.org/10.5194/aab-68-485-2025-supplement.

## Data Availability

The relevant data are provided in Table 2 to 6 and in the Supplement. The data from the current experiment can be obtained from the corresponding author when needed.
