# Supplementary material for: Enzyme inclusion or fermentation of canola-based diets generate different responses in growth indicators, carcass quality, nutrient digestibility, bone strength, and blood biochemical parameters in broiler chickens
Source: Arch Anim Breed. 2025 Jul 25;68(3):485–95. doi: 10.5194/aab-68-485-2025 (PMC13463655; doi:10.5194/aab-68-485-2025)
Supplement: Supplementary file 1 [file aab-68-485-2025-supplement.pdf]

Supplement of Arch. Anim. Breed., 68, 485–495, 2025
https://doi.org/10.5194/aab-68-485-2025-supplement
© Author(s) 2025. CC BY 4.0 License.

Open Access
Archives Animal Breeding

Supplement of

Enzyme inclusion or fermentation of canola-based diets generate different responses in growth indicators, carcass quality, nutrient digestibility, bone strength, and blood biochemical parameters in broiler chickens

Abdul Hafeez et al.

Correspondence to: Shabana Naz (drshabananaz@gcuf.edu.pk) and Ibrahim A. Alhidary (ialhidary@ksu.edu.sa)

The copyright of individual parts of the supplement might differ from the article licence.

Table S1 - Interaction effect of dietary enzyme treatment and fermentation on Feed Intake (g) in broilers											
**Groups**	**UC6**	**UC12**	**UC18**	**EC6**	**EC12**	**EC18**	**FC6**	**FC12**	**FC18**	**SEM**	**P-Value**
WK1	140[a]	120[b]	118[b]	142[a]	136[a]	118[b]	142[a]	137[a]	118[b]	2.18	0.00
WK2	290	291	280	287	279	282	291	289	273	2.41	0.63
WK3	510[ab]	503[ab]	512[ab]	544[a]	486[b]	502[ab]	516[ab]	489[ab]	494[ab]	4.64	0.07
Starter	940[ab]	914[ab]	910[ab]	973[a]	900[ab]	903[ab]	949[ab]	915[ab]	884[b]	6.83	0.03
WK4	773[ab]	778[ab]	772[ab]	805[a]	720[b]	743[ab]	794[ab]	750[ab]	786[ab]	6.55	0.02
WK5	859	851	814	865	835	850	854	847	811	6.22	0.42
WK6	931	917	910	958	911	901	950	923	898	5.33	0.05
Finisher	2563	2546	2496	2627	2465	2494	2598	2520	2495	13.9	0.06
Overall	3504[ab]	3460[ab]	3406[ab]	3601[a]	3365[b]	3396[b]	3547[ab]	3435[ab]	3379[b]	18.6	0.01

Means in the same row with different superscripts are significantly different at $\alpha = 0.05$

UC6: Untreated Canola at 6%
UC12: Untreated Canola at 12%
UC18: Untreated Canola at 18%
EC6: Enzyme Treated Canola at 6%
EC12: Enzyme Treated Canola at 12%
EC18: Enzyme Treated Canola at 18%
FC6: Fermented Canola at 6%
FC12: Fermented Canola at 12%
FC18: Fermented Canola at 18%

Table S2 - Interaction effect of dietary enzyme treatment and fermentation on Weight Gain (g) in broilers											
**Groups**	**UC6**	**UC12**	**UC18**	**EC6**	**EC12**	**EC18**	**FC6**	**FC12**	**FC18**	**SEM**	**P-Value**
WK1	126[a]	105[b]	106[b]	123[a]	122[a]	101[b]	121[a]	127 [a]	101[b]	2.20	<0.01
WK2	253[a]	241[ab]	230[ab]	246[ab]	245[ab]	238[ab]	249 [ab]	257 [a]	221 [b]	2.64	0.01
WK3	345[ab]	313[bc]	314[bc]	333[abc]	356[a]	305[c]	336[abc]	358[a]	316[bc]	4.18	<0.01
Starter	724[a]	659[bcd]	650[cd]	702[abc]	723[a]	644[d]	706[ab]	743[a]	638[d]	7.91	<0.01
WK4	401[ab]	379[bc]	374[bc]	402[ab]	418[a]	369[c]	401[ab]	418[a]	373[bc]	4.00	<0.01
WK5	357[abc]	332[abc]	313[c]	350[abc]	373[a]	323[bc]	356[abc]	371 [ab]	317 [c]	5.07	<0.01
WK6	371[a]	346[bc]	343[bc]	371[a]	361[abc]	343[bc]	367 [ab]	374[a]	341[c]	2.93	<0.01
Finisher	1129[a]	1056[bc]	1030[c]	1123[ab]	1152[a]	1035[c]	1124[ab]	1163[a]	1031[c]	10.90	<0.01
Overall	1852[a]	1716[b]	1680[b]	1825[a]	1874[a]	1679[b]	1830[a]	1906[a]	1669[b]	18.27	<0.01

Means in the same row with different superscripts are significantly different at $\alpha = 0.05$

UC6: Untreated Canola at 6% UC12: Untreated Canola at 12% UC18: Untreated Canola at 18%
EC6: Enzyme Treated Canola at 6% EC12: Enzyme Treated Canola at 12% EC18: Enzyme Treated Canola at 18%
FC6: Fermented Canola at 6% FC12: Fermented Canola at 12% FC18: Fermented Canola at 18%

Table S3 - Interaction effect of dietary enzyme treatment and fermentation on Feed Conversion Ratio in broilers										
**Groups**	**UC6**	**UC12**	**UC18**	**EC6**	**EC12**	**EC18**	**FC6**	**FC12**	**FC18**	**SEM**	**P-Value**
WK1	1.11	1.15	1.12	1.15	1.12	1.17	1.17	1.07	1.17	.009	0.60
WK2	1.15	1.21	1.22	1.17	1.14	1.19	1.17	1.13	1.23	.013	0.53
WK3	1.49^{ab}	1.61^{ab}	1.63^{ab}	1.64^{a}	1.37^{b}	1.65^{a}	1.54^{ab}	1.37^{b}	1.56^{ab}	.025	0.05
Starter	1.30^{abc}	1.39^{a}	1.40^{a}	1.39^{a}	1.25^{bc}	1.40^{a}	1.35^{abc}	1.23^{c}	1.39^{ab}	.015	0.01
WK4	1.94^{abc}	2.06^{ab}	2.07^{ab}	2.00^{abc}	1.72^{c}	2.01^{ab}	1.98^{abc}	1.79^{bc}	2.11^{a}	.029	0.02
WK5	2.42	2.57	2.61	2.48	2.24	2.63	2.40	2.28	2.57	.038	0.95
WK6	2.51^{ab}	2.65^{a}	2.66^{a}	2.58^{ab}	2.52^{ab}	2.63^{ab}	2.59^{ab}	2.47^{b}	2.63^{a}	.016	0.05
Finisher	2.28^{ab}	2.41^{a}	2.42^{a}	2.34^{ab}	2.14^{b}	2.41^{a}	2.31^{ab}	2.16^{b}	2.42^{a}	.024	0.01
Overall	1.90^{ab}	2.02^{a}	2.03^{a}	1.97^{a}	1.80^{b}	2.02^{a}	1.94^{ab}	1.80^{b}	2.03^{a}	.019	<0.01

Means in the same row with different superscripts are significantly different at $\alpha = 0.05$

Table S4 - Interaction effect of dietary enzyme treatment and fermentation on percent Apparent Total Digestibility (ATD) of nutrients at finisher phase in broilers

Groups	UC6	UC12	UC18	EC6	EC12	EC18	FC6	FC12	FC18	SEM	P-Value
DM	72.6	72.6	73.4	71.9	74.7	71.0	71.5	74.6	75.5	0.43	0.30
ASH	46.5	47.9	45.3	48.2	48.2	47.3	48.0	48.7	46.8	0.41	0.68
CP	70.3^{ab}	60.0^{c}	59.8^{c}	59.7^{c}	73.9^{a}	61.6^{c}	70.9^{a}	72.6^{a}	65.1^{bc}	0.99	<0.01
CF	78.4^{a}	70.8^{b}	68.5^{b}	70.7^{b}	79.6^{a}	67.5^{b}	78.8^{a}	79.2^{a}	74.4^{ab}	0.90	<0.01
NFE	83.1^{abcd}	79.3^{d}	80.1^{cd}	78.6^{d}	85.5^{ab}	79.8^{d}	85.1^{abc}	86.0^{a}	80.4^{bcd}	0.57	<0.01
Ca	28.1^{ab}	24.7^{b}	24.6^{b}	26.2^{ab}	29.9^{a}	27.1^{ab}	24.9^{b}	29.9^{a}	24.9^{b}	0.43	<0.01
P	29.2^{ab}	24.5^{ab}	23.8^{b}	24.8^{ab}	30.3^{a}	23.2^{b}	24.9^{ab}	29.2^{ab}	24.1^{b}	0.58	0.01

Means in the same row with different superscripts are significantly different at $\alpha = 0.05$

UC6: Untreated Canola at 6% UC12: Untreated Canola at 12% UC18: Untreated Canola at 18%

EC6: Enzyme Treated Canola at 6% EC12: Enzyme Treated Canola at 12% EC18: Enzyme Treated Canola at 18%

FC6: Fermented Canola at 6% FC12: Fermented Canola at 12% FC18: Fermented Canola at 18%

Table S5 - Interaction effect of dietary enzyme treatment and fermentation on bone quality in broilers											
**Groups**	**UC6**	**UC12**	**UC18**	**EC6**	**EC12**	**EC18**	**FC6**	**FC12**	**FC18**	**SEM**	**P-Value**
Bone Weight (g)	7.86^{ab}	5.67^{b}	5.68^{b}	6.48^{ab}	8.15^{ab}	5.33^{b}	8.89^{a}	8.98^{a}	6.52^{ab}	0.31	<0.01
Bone Length (cm)	90.5	89.0	93.5	92.6	89.2	88.9	86.4	86.8	87.0	0.76	0.29
BW: BoneWt	246	255	248	251	256	249	255	246	254	1.54	0.71
Robusticity Index	4.56^{abc}	5.00^{ab}	5.24^{a}	4.97^{ab}	4.43^{bc}	5.11^{ab}	4.17^{c}	4.18^{c}	4.73^{abc}	0.08	<0.01
Tibio Tarsal Index	44.0	38.5	37.2	35.9	38.4	35.9	34.1	27.8	32.4	1.26	1.66

Means in the same row with different superscripts are significantly different at α= 0.05

UC6: Untreated Canola at 6% UC12: Untreated Canola at 12% UC18: Untreated Canola at 18%

EC6: Enzyme Treated Canola at 6% EC12: Enzyme Treated Canola at 12% EC18: Enzyme Treated Canola at 18%

FC6: Fermented Canola at 6% FC12: Fermented Canola at 12% FC18: Fermented Canola at 18%

Table S6 - Interaction effect of dietary enzyme treatment and fermentation on carcass characteristics in broilers

Groups	UC6	UC12	UC18	EC6	EC12	EC18	FC6	FC12	FC18	SEM	P-Value
Dressing %	68.97[a]	62.6[cd]	61.7[d]	65.7[abc]	68.43[ab]	66.3[ab]	66.9[ab]	68.97[a]	65.4[bc]	0.52	<0.01
Eviscerated Weight (%)	77.21[abc]	71.7[c]	71.5[c]	78.8[abc]	78.60[abc]	73.3[bc]	81.2[ab]	82.34[a]	77.1[abc]	0.85	<0.01
Giblet Weight (g)	76.00	96.7	75.3	74.3	84.33	75.3	79.0	77.67	80.3	1.82	0.06
Abdominal Fat Weight (g)	1.67	1.67	1.91	1.53	1.70	1.84	1.55	1.61	1.73	0.04	0.30
Meat pH	5.97	5.97	5.97	6.00	5.97	5.97	5.97	5.97	5.97	0.01	1.00

Means in the same row with different superscripts are significantly different at α= 0.05

UC6: Untreated Canola at 6% UC12: Untreated Canola at 12% UC18: Untreated Canola at 18%
EC6: Enzyme Treated Canola at 6% EC12: Enzyme Treated Canola at 12% EC18: Enzyme Treated Canola at 18%
FC6: Fermented Canola at 6% FC12: Fermented Canola at 12% FC18: Fermented Canola at 18%

Table S7- Interaction effect of dietary enzyme treatment and fermentation on blood biochemical parameters at finisher phase in broilers											
	**UC6**	**UC12**	**UC18**	**EC6**	**EC12**	**EC18**	**FC6**	**FC12**	**FC18**	**SEM**	**P-value**
Triglycerides (mg/dL)	42.1	41.9	40.5	39.8	39.2	41.6	39.2	38.6	36.9	0.45	0.08
LDL (mg/dL)	40.4	37.2	36.2	37.9	42.4	37.1	41.4	38.3	40.0	0.72	0.48
HDL (mg/dL)	55.1	54.8	59.2	61.1	54.0	55.5	56.1	60.3	58.1	0.71	0.13
Total Cholesterol (mg/dL)	103.9	100.4	103.4	107.0	104.3	100.9	105.3	106.2	105.6	0.91	0.76
Blood Glucose (mg/dL)	111.6	111.7	111.8	111.7	111.6	111.9	111.3	112.3	111.8	0.83	0.87
Total Protein (g/dL)	4.50	4.37	3.90	4.37	4.17	4.03	3.97	4.43	4.60	0.12	0.93

UC6: Untreated Canola at 6% UC12: Untreated Canola at 12% UC18: Untreated Canola at 18%
EC6: Enzyme Treated Canola at 6% EC12: Enzyme Treated Canola at 12% EC18: Enzyme Treated Canola at 18%
FC6: Fermented Canola at 6% FC12: Fermented Canola at 12% FC18: Fermented Canola at 18%
